# Thoracic perfusion of matrine as an adjuvant treatment improves the control of the malignant pleural effusions

**DOI:** 10.1186/s12957-015-0729-9

**Published:** 2015-12-03

**Authors:** Rong Biaoxue, Ma Shuxia, Gao Wenlong, Yang Shuanying

**Affiliations:** Department of Respiratory Medicine, The Second Affiiated Hospital, Xi’an Jiaotong University, Xi’an, China; Department of Elderly Respiratory Medicine, The Second Affiliated Hospital, Xi’an Jiaotong University, Xi’an, China; Department of Statistics and Epidemiology, Medical College, Lanzhou University, Lanzhou, China

**Keywords:** Matrine, Malignant pleural effusion, Therapy, Quality of life, Meta-analysis

## Abstract

**Background:**

Many studies have investigated the efficacy and safety of matrine in treating malignant pleural effusion by thoracic perfusion. This study is an analytic value of available evidence.

**Methods:**

Twelve studies were analyzed in this study. Pooled odds ratios and hazard ratio with 95 % confidence intervals were calculated using the fixed effects model.

**Results:**

Overall response rate of matrine combined with other medications in treating malignant pleural effusion (MPE) was significantly higher than those of other medications alone (*p* < 0.05). Time to pleural effusion relief and quality of life were improved after the treatment of matrine combined with other medications (*p* < 0.05). Moreover, matrine combined with other medications had a lower incidence of adverse reactions (*p* < 0.05).

**Conclusions:**

Matrine combined with other medications improves the control of the malignant pleural effusions and decreases the incidence of adverse reactions.

## Background

Malignant pleural effusion (MPE) poses a significant clinical problem. In oncology patients, there are a number of common medical problems associated with the development of pleural effusion which frequently coexist with the malignancy. Tumor-induced impairment of pleural fluid drainage and pertinent findings point toward another pathway to MPE formation: a vicious loop of interactions between pleural-based tumor cells and the host vasculature and immune system that results in increased net fluid production via enhanced plasma extravasation into the pleural space [1]. As a result, patients with MPE face a limited survival of a few months, depending on the underlying malignancy. First-line treatment of MPE may include chemotherapy aimed at tumor shrinkage and pleural fluid absorption. However, most causative tumors are or become chemoresistant, and many patients with MPE are not fit for chemotherapy. Therefore, treatment commonly relies on palliative measures aimed at improving quality of life [1]. Therefore, novel, effective, safe, and convenient treatment modalities for patients with MPE are needed. Nowadays, novel strategy and effective therapies that were needed to improve outcome of these patients remain challenging. Nowadays, to discover and develop novel natural compounds that have therapeutic selectivity or that can preferentially kill lung cancer cells without significant toxicity to normal cells is an important tendency for therapy of MPE. Accumulating research evidence suggests that many medicinal plants may be used alone or in combination with common chemotherapeutic agents to treat MPE. Due to their wide range of biological activities and low toxicity in animal models, these products have been used as alternative treatments for MPE.

Matrine is a naturally occurring small-molecule compound from traditional Chinese medicine *Sophora flavescens* Ait. In China, matrine as a clinical drug has been used to treat cancer. The results indicated that matrine induced the apoptosis of murine hepatoma cells in vitro and in vivo as well as inhibited tumor growth [2] and also inhibited the invasiveness and metastasis of human malignant melanoma cell line A375 [3]. Some studies reported that matrine induced gastric cancer MKN45 cell apoptosis [4] and reduced Hela cell adhesion and migration [5]. Matrine was approved by the China State Food and Drug Administration (SFDA) for the treatment of cancer in 1992. To date, some studies discuss the efficacy and safety of murine in treating MPE by thoracic perfusion. Whether or not matrine has the potential therapeutic and/or adjuvant therapeutic application in the treatment of human MPE is conflicting. This study presents a systematic study to quantify the toxicities and clinical benefits of matrine combined with other medications versus other medications alone in treating advanced MPE.

## Methods

### Search strategy and data extraction

An electronic search of scientific literature published in the databases of MEDLINE/PubMed, Embase, Cochrane Library, Science Citation Index, and CNKI was performed using free text and Medical Subject Heading terms such as “malignant pleural effusion,” “MPE,” “matrine,” “oxymatrine,” “matrine injection,” “kushenzongjian zhusheye,” “fufang kushen zhusheye,” “chemotherapy,” “sophora flavescens ait,” and “shrubby sophora extract.” The search period was from the start of each database up to May 2015 without language restrictions. Moreover, a manual revision of the bibliographical references of the selected articles was done. The extracted data are summarized as follows: (1) general information, including the title, author, publication date, and literature sources; (2) design and implementation, including the type of design, research and follow-up time, interventions, measurement indicator, the number of lost and processed samples; and (3) outcome indicators, including response rate (RR), disease control rate (DCR), mean survival time (MST), time to progression (TTP), quality of life (QOL), and adverse effects (AEs).

### Criteria for inclusion and exclusion

Inclusion criteria were as follows: (1) trials must compare matrine combined with other medications to other medications alone through thoracic perfusion; (2) patients in the studies must be diagnosed and confirmed by cytology and pathology; (3) age and gender must not be restricted; (4) must report on at least one of the outcome measures mentioned in the succeeding portion of this study; and (5) the total number of cases must be greater than or equal to 80. The following studies were excluded: (1) those with no clearly reported outcomes of interest; (2) studying on animals not on human; and (3) studies lacking control groups.

### Type of trial design, interventions, and indicators to determine efficacy

Trial design: randomized controlled trials of matrine combined with other medications versus other medications alone in treating MPE by thoracic perfusion. Type of interventions: matrine + other medications vs. other medications alone; efficacy indicators: ORR, DCR, MST, TTP, QOL, and AEs (according to the toxicity criteria of WHO).

### Methodological quality assessment

The methodological quality for RCTs was assessed using the criteria from the Cochrane Handbook for Systematic Reviews of Interventions (version 5.0.1). The quality of trials was categorized into low risk of bias, unclear risk of bias, or high risk of bias. This categorization was according to the risk for each important outcome within included trials, including adequacy of the generation of allocation sequence, allocation concealment, blinding, and the presence of incomplete outcome data, selective outcome, or other sources of bias. The intention-to-treat (ITT) analysis was also assessed for the randomized controlled trials included into the present meta-analysis [6, 7].

### Statistical analysis

To assess the efficacy and safety of matrine combined with other medications versus other medications alone for treating MPE, fixed effects model was performed. Dichotomous variables were analyzed using estimation of odds ratios (OR) with a 95 % confidence interval (95 % CI). The overall effect was tested using *Z*-scores, with significance being set at *p* < 0.05. Pooled effect was calculated using either the fixed effects model or random effects model. Heterogeneity was evaluated through chi-square and *I*^2^. Meta regression was done to evaluate whether results were different between two groups. Sensitivity was analyzed by omitting each study from the estimated pool conducted at each step. Finally, publication bias was evaluated using funnel plots, the Egger’s test, and the Begg’s test. Statistical analyses were performed using SPSS (SPSS Institute, version 19.0, Chicago, USA), RevMan 5.2 (The Cochrane Collaboration), and Stata version 13.0 (Stata Corporation, TX, USA). All *p* values were two-sided, and *p* < 0.05 was considered to indicate statistical significance.

## Results

### Selection of studies

**Fig. 1:**
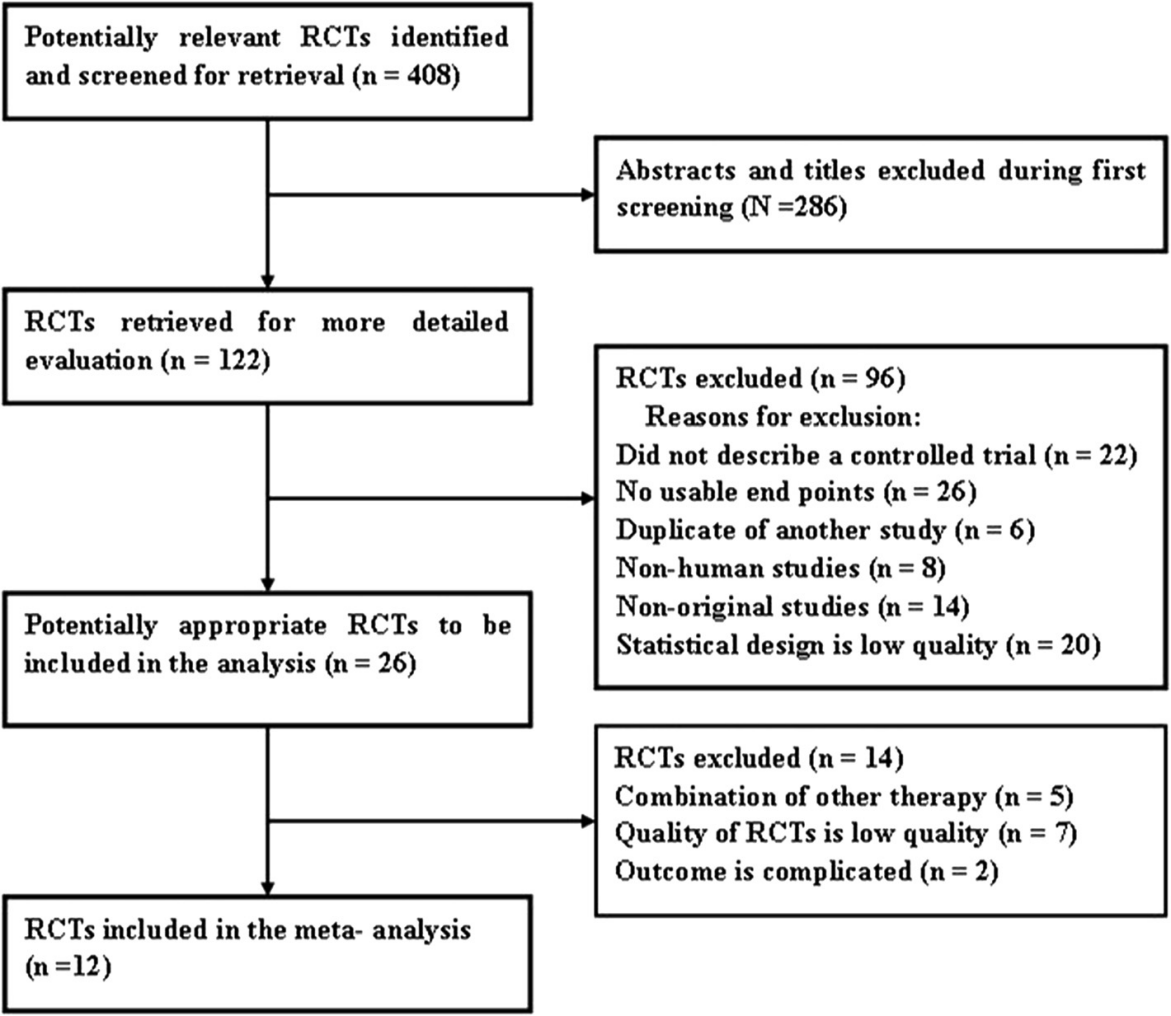
Flow chart of literature search. *RCTs* randomized controlled trials

Our systematic search identified 408 potentially relevant abstracts, of which 122 were identified as requiring full-text article retrieval. Close screening of these 122 studies excluded 109 because of the following reasons: limited cases, non-human studies, and some received matrine therapy without a parallel control. Finally, 12 studies2 [8–19] published between 2006 and 2014 matched the inclusion criteria and were therefore included (Fig. 1). Table 1 shows the baseline demographic factors of the patients. The eligible studies included 1320 patients, and the sample sizes oscillated between 80 [8] and 168 [15] patients, and the age of the patients mainly concentrated at the range of 40 to 70 years old, with the youngest at 20 years old [8] and the oldest at 85 years old [15].

**Table 1:** Patient characteristics of the clinical trials reviewed

Study	Number of patients	M/F	Age	Sources of tumor (*N*)	Volume of MPE (*N*)	Quality of life	Pleural perfusion (*N*)	Group 1/2 (*N*)	End point
Yunfang et al. [15]	168	NA	38–85	Lung/pleura (102)	NA	KPS	P + M versus P	84/84	RR, DCR, SI, AEs
				Breast (32)					
				Digestive tract (34)					
Sijie et al. [13]	153	68/85	32–84	Lung/pleura (117)	Large (57)	KPS	IL-11 + P + M versus IL-11 + P	75/78	RR, DCR, SI, AEs
				Breast (26)	Moderate (82)				
				Lymphoma (10)	Small (14)				
Zenmin et al. [9]	110	NA	35–83	Lung/pleura (61)	NA	KPS	P + M versus P	56/54	RR, DCR, SI, AEs
				Breast (32)					
				Digestive tract (17)					
Daiju and Xiaodong [19]	150	96/54	45-75	Lung/pleura (150)	NA	KPS	IL-2 + M versus IL-2	75/75	RR, DCR, SI, AEs
							а-IFN + M versus a-IFN		
Zhiwen et al. [12]	90	47/43	40–77	Lung (90)	NA	KPS	CP + M versus CP	45/45	RR, DCR, SI, AEs
Liangfa et al. [11]	98	56/42	48–73	Lung/pleura (58)	Large (57)	KPS	P + M versus P	50/48	RR, DCR, SI, AEs
				Breast (24)	Moderate (23)				
				Digestive tract (6)	Small (18)				
				Lymphoma (7)					
				Others (3)					
Xiaowei et al. [10]	120	67/53	49–76	Lung/pleura (49)	NA	KPS	MMC + M versus MMC	60/60	RR, DCR, SI, AEs
				Breast (19)					
				Digestive tract (32)					
				Lymphoma (8)					
				Others (12)					
Guoan [18]	86	54/32	65-81	Lung(86)	NA	KPS	BLM + M versus BLM	44/42	RR, DCR, SI, AEs
Li [8]	80	46/34	20–82	Lung/pleura (29)	Large (48)	KPS	P + M versus P	40/40	RR, DCR, SI, AEs
				Breast (14)	Moderate (32)				
				Digestive tract (21)					
				Lymphoma (7)					
				Others (2)					
Ziqiang [14]	90	57/33	31–75	Lung/pleura (43)	NA	KPS	IL-2 + M versus IL-2 + P	45/45	RR, DCR, SI, AEs
				Breast (20)					
				Digestive tract (18)					
				Others (9)					
Yushun and Jie [16]	93	63/30	NA	Lung/pleura (93)	NA	KPS	P + M versus P	47/46	RR, DCR, SI, AEs
Zhenfeng et al. [17]	82	50/36	60–82	Lung (82)	NA	KPS	HCPT + M versus HCPT	42/40	RR, DCR, SI, AEs

*Abbreviations: M/F* male/ female, *MPE* malignant pleural effusion, *Group 1* matrine combined with other therapy, *Group 2* other therapy alone, *KPS* Karnofsky physical status score, *NA* not available, *P* cisplatin, *M* murine, *RR* response rate, *DCR* disease control rate, *SI* symptom improvement, *AEs* adverse effects, *IL-11* interleukin-11, *IL-2* interleukin-2, *а-IFN* а-interferon, *CP Corynebacterium parvum*, *MMC* mitomycin C, *BLM* bleomycin, *HCPT* hydroxycamptothecin

### Quality of study design

The studies were appraised independently by three authors (Rong BX, Ma SX, and Gao WL) based on the criteria from the Cochrane Handbook for Systematic Reviews of Interventions (version 5.0.1). According to our predefined quality assessment criteria, 9 of the 12 trials (75 %) were evaluated as having a low risk-of-bias, and another 3 included trials were evaluated as having an unclear risk-of-bias (25 %). Table 2 shows the quality of each study included in the present systematic review.

**Table 2:** Raw data and methodological quality of included trials

Studies	Region	Sequence generation	Allocation concealment	Blind	Outcome data	Selective outcome reporting	Other sources of bias	ITT	Risk of bias
Yunfang et al. [15]	Single center	Random number table (SAS)	Insufficient	Clear	No	No	Unclear	Yes	Low risk of bias
Sijie et al. [13]	Single center	Random number table (SPSS)	Unclear	Unclear	Yes	No	Clear	No	Unclear risk of bias
Zenmin et al. [9]	Single center	Random number table (SPSS)	Insufficient	Unclear	Yes	No	Unclear	No	Low risk of bias
Daiju and Xiaodong [19]	Single center	unclear	Unclear	Clear	Yes	No	Unclear	No	Unclear risk of bias
Zhiwen et al. [12]	Single center	Random number table (SPSS)	Unclear	Unclear	Yes	No	Clear	No	Unclear risk of bias
Liangfa et al. [11]	Single center	Random number table (SPSS)	Clear	Clear	Yes	No	Clear	No	Unclear risk of bias
Xiaowei et al. [10]	Single center	Random number table (SPSS)	Insufficient	Unclear	Yes	No	Clear	No	Unclear risk of bias
Guoan [18]	Single center	Random number table (SPSS)	Clear	Unclear	Yes	Yes	Clear	No	Unclear risk of bias
Li [8]	Single center	Random number table (SAS)	Insufficient	Unclear	Yes	No	Clear	No	Unclear risk of bias
Ziqiang [14]	Single center	Random number table (SPSS)	Unclear	Unclear	Yes	No	Unclear	No	Unclear risk of bias
Yushun and Jie [16]	Single center	Random number table (SPSS)	Insufficient	Clear	Yes	No	Unclear	No	Low risk of bias
Zhenfeng et al. [17]	Single center	Random number table (SPSS)	Clear	Clear	Yes	No	Clear	No	Unclear risk of bias

*SAS* SAS software, *SPSS* SPSS software, *ITT* intention-to-treat

### Comparison of ORR between matrine combined with other medications and other medications alone

**Fig. 2:**
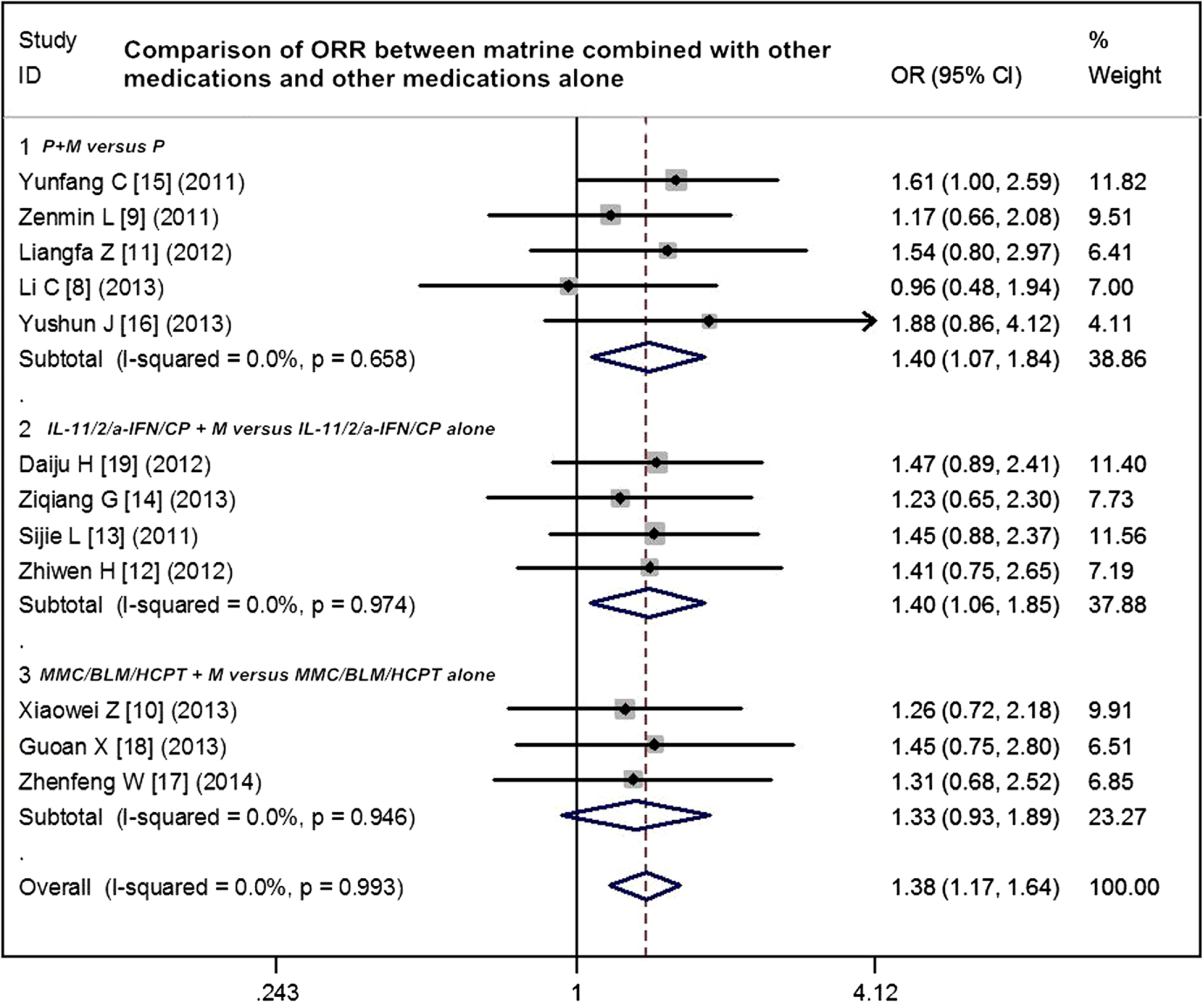
Comparison of ORR between matrine combined with other medications and other medications alone. *ORR* overall response rate, *OR* odds ratio, *P* cisplatin, *M* murine, *IL-11* interleukin-11, *IL-2* interleukin-2, *а-IFN* а-interferon, *CP Corynebacterium parvum*, *MMC* mitomycin C, *BLM* bleomycin, *HCPT* hydroxycamptothecin

Twelve studies compared the ORR between matrine combined with other medications and other medications alone for MPE. The results of the fixed effects model showed that OR = 1.38 (95 % CI 1.17 to 1.64; test for heterogeneity =3.78; *I*^2^ = 0 %) and test for overall effect *Z* = 3.04, *p* = 0.002. The ORR of matrine combined with other medications was significantly higher than that of with other medications alone. The subgroup analyses showed that ORR favored the following three matrine combinations with the overall effect *Z*-value and *p* values as follows: cisplatin + matrine versus cisplatin alone (*Z* = 1.31, *p* = 0.018); biological agents (including interleukin-11 (IL-11), interleukin-2 (IL-2), а-interferon (а-IFN), and *Corynebacterium parvum*) + matrine versus biological agents alone (*Z* = 2.40, *p* = 0.016); and other chemotherapeutic agents (including mitomycin C (MMC), bleomycin (BLM), and hydroxycamptothecin (HCPT)) + matrine versus chemotherapeutic agents alone (*Z* = 1.55, *p* = 0.012) (Fig. 2). Sensitivity analyses showed that the RR and 95 % CI did not alter substantially by removing any one trial (data not shown), with an OR pool oscillating between 0.96 and 1.88.

### Comparison of QOL between matrine combined with other medications and other medications alone

**Fig. 3:**
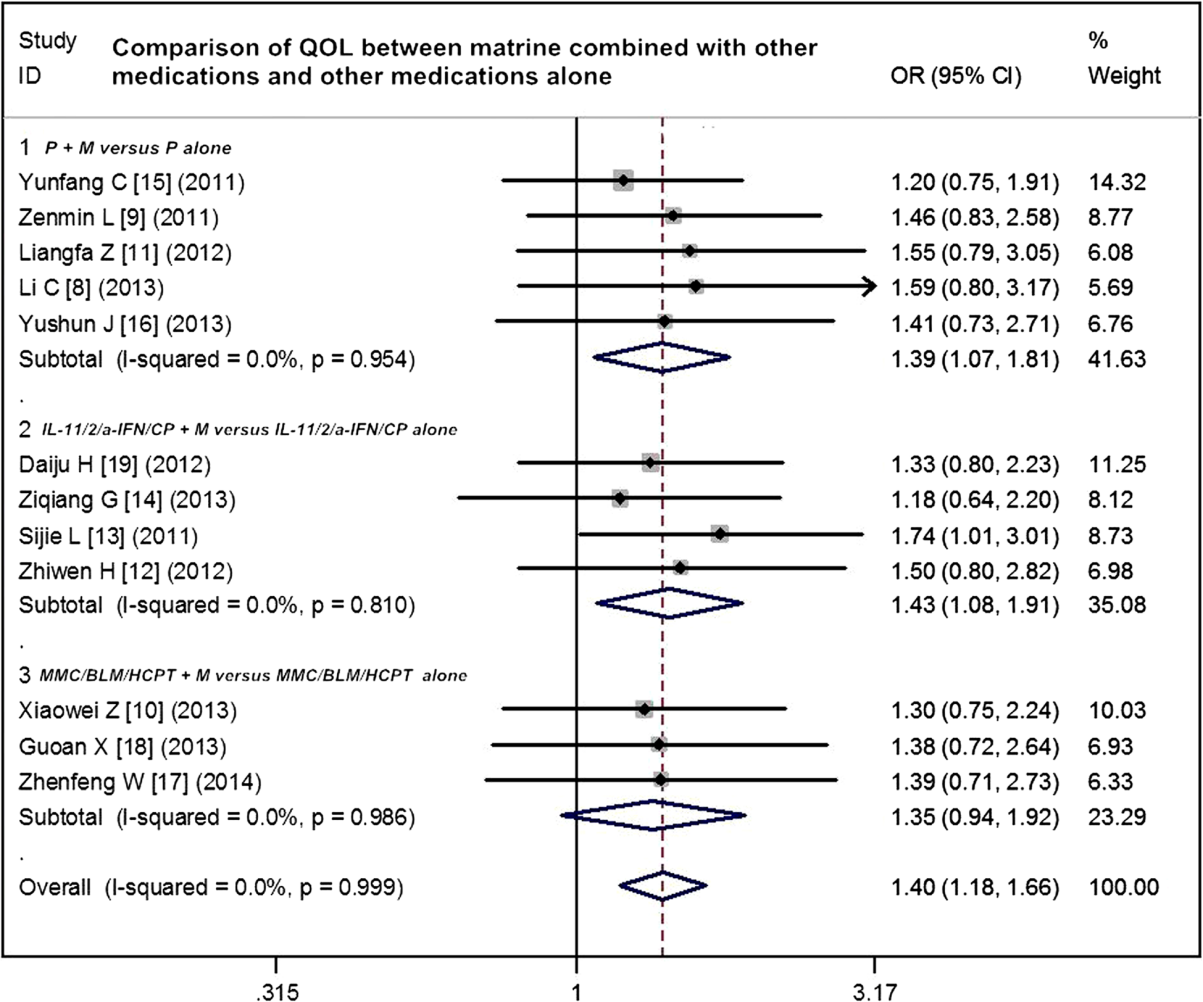
Comparison of QOL between matrine combined with other medications and other medications alone. *ORR* overall response rate, *OR* odds ratio, *P* cisplatin, *M* murine, *IL-11* interleukin-11, *IL-2* interleukin-2, *а-IFN* а-interferon, *CP Corynebacterium parvum*, *MMC* mitomycin C, *BLM* bleomycin, *HCPT* hydroxycamptothecin

Twelve trials compared the QOL between matrine combined with other medications and other medications alone for MPE. The results of the fixed effects model showed that OR = 1.40 (95 % CI 1.18 to 1.66; test for heterogeneity =1.74; *I*^2^ = 0 %) and test for overall effect *Z* = 3.84, *p* < 0.0001. Matrine combined with other medications significantly improves the QOL of MPE patients. The subgroup analyses showed that ORR favored the following three matrine combinations with the overall effect *Z*-value and *p* values as follows: cisplatin + matrine versus cisplatin alone (*Z* = 2.46, *p* = 0.014); biological agents (including IL-11, IL-2, а-IFN, and *Corynebacterium parvum*) + matrine versus biological agents alone (*Z* = 2.48, *p* = 0.013); and other chemotherapeutic agents (including MMC, BLM, and HCPT) + matrine versus chemotherapeutic agents alone (*Z* = 1.65, *p* = 0.100) (Fig. 3). In the analysis of sensitivity, the exclusion of studies individually did not substantially modify the estimators, with an OR pool oscillating between 1.20 and 1.74.

### Comparison of time to pleural effusion relief between matrine combined with other medications and other medications alone

**Fig. 4:**
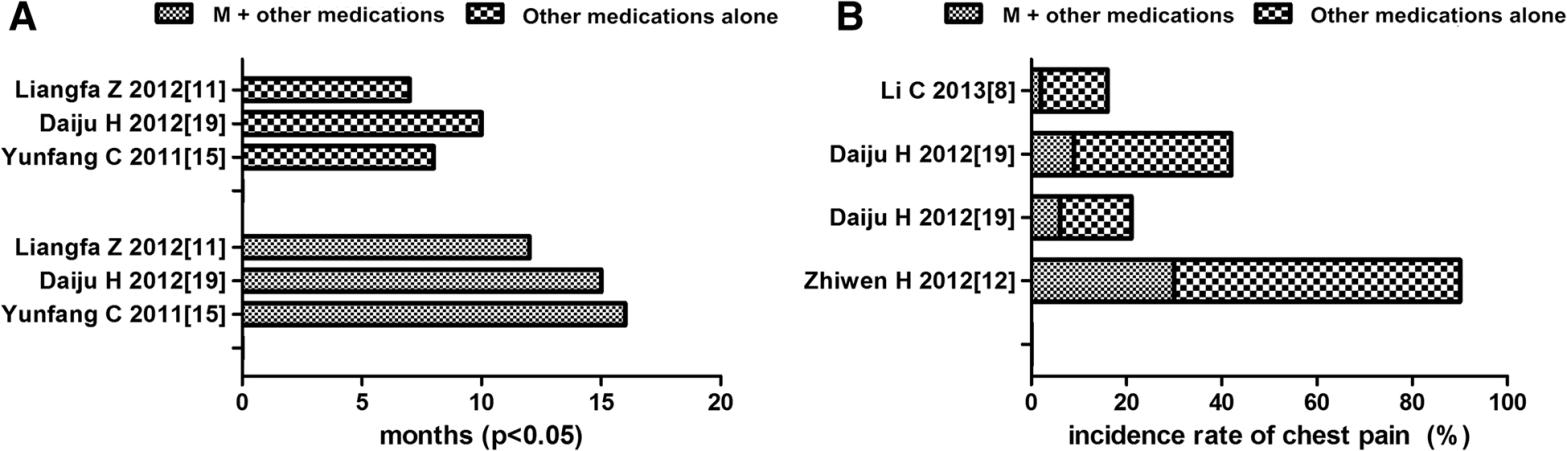
Comparison of time to pleural effusion relief and chest pain between matrine combined with other medications and other medications alone. **a** Comparison of time to pleural effusion relief between matrine combined with other medications and other medications; **b** comparison of chest pain between matrine combined with other medications and other medications alone

Three studies reported time to pleural effusion relief, and the results showed that the time to pleural effusion relief (mean ± SD) of matrine combined with other medications and other medications alone was 14.33 ± 1.20 and 8.33 ± 0.88 months, respectively. The *t* value was 4.025; the degrees of freedom was 3, *p* = 0.015 (Table 3). The time to pleural effusion relief of matrine combined arm was significantly longer than that of other medications alone (Fig. 4a).

**Table 3:** Comparison of time to pleural effusion relief of matrine combined with other medications and other medications alone

	Matrine combined with other medications (months)	Other medications alone (months)	*T*-value	95 % CI	*p* value
Yunfang et al. [15]	16.4	8.2	*T* = 4.025	1.862 to 10.14	0.015
Daiju and Xiaodong [19]	15.98	10.06			
			*df* = 3		
Liangfa et al. [11]	12.00	7.00			
Mean ± SD	14.33 ± 1.20	8.33 ± 0.88			

*95 % CI* 95 % confidence interval, *SD* standard deviation

### Comparison of chest pain between matrine combined with other medications and other medications alone

Four trials conducted a statistical analysis of the change of chest pain between matrine combined with other medications and other medications alone. The result indicated that the chest pain was significantly decreased by treatment of matrine combined with other medications than that of other medications alone (*p* < 0.05) (Table 4, Fig. 4b).

**Table 4:** Comparison of chest pain between matrine combined with other medications and other medications alone

	Matrine combined with other medications (%)	Other medications alone (%)	*T*-value	95 % CI	*p* value
Zhiwen et al. [12]	15 (33.3)	27 (60)	*T* = 3.781	2.862 to 9.14	0.006
Xiaowei et al. [10]	4 (6)	9 (15)	*df* = 4		
Daiju and Xiaodong [19]	7 (9)	25 (33)			
Li [8]	1 (2)	7 (14)			
Overall incidence	27 (12)	68 (31)			

*95 % CI* 95 % confidence interval

### Adverse reactions analysis of matrine combined with other medications and other medications alone

**Fig. 5:**
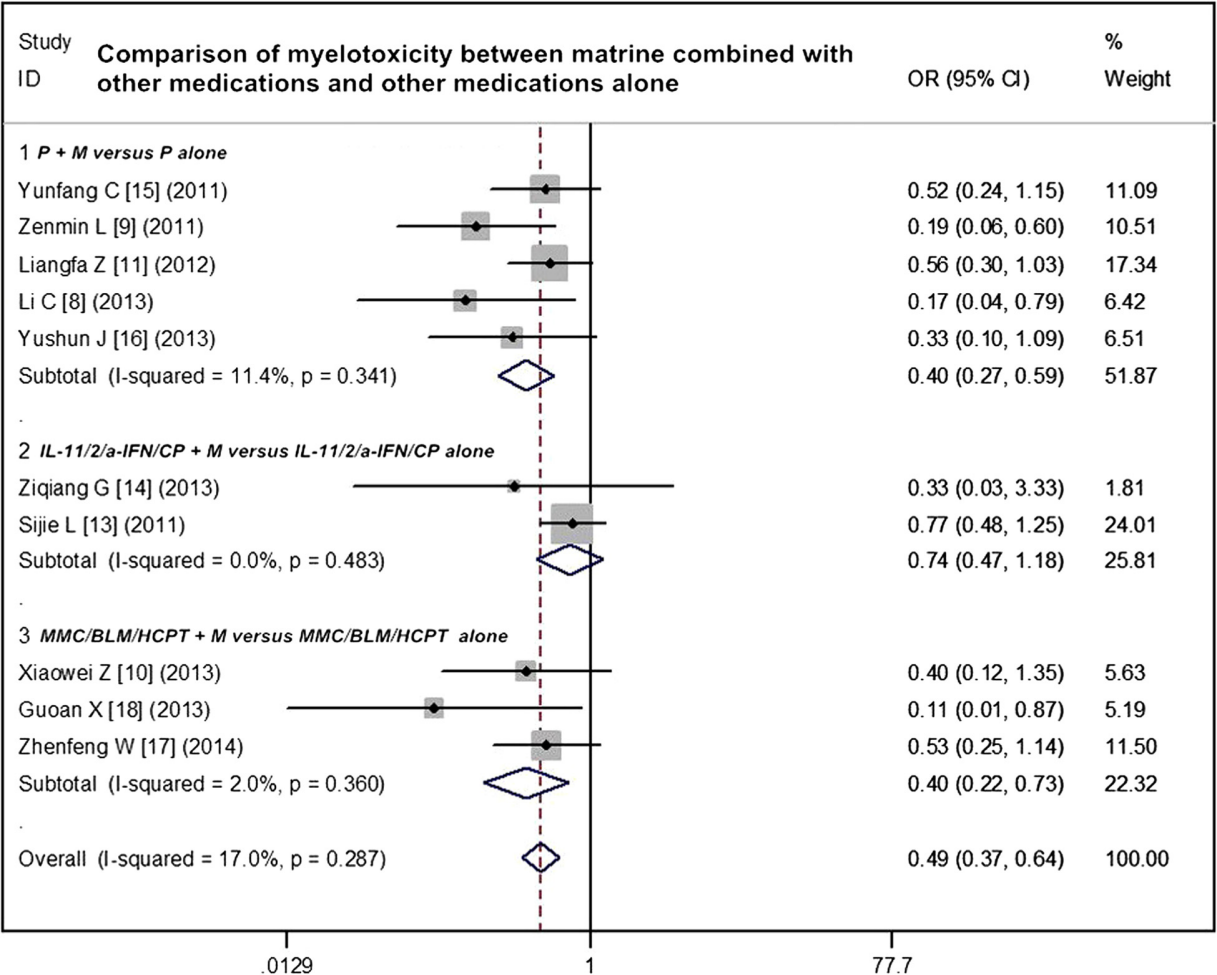
Comparison of myelotoxicity between matrine combined with other medications and other medications alone. *ORR* overall response rate, *OR* odds ratio, *P* cisplatin, *M* murine, *IL-11* interleukin-11, *IL-2* interleukin-2, *а-IFN* а-interferon, *CP Corynebacterium parvum*, *MMC* mitomycin C, *BLM* bleomycin, *HCPT* hydroxycamptothecin

**Fig. 6:**
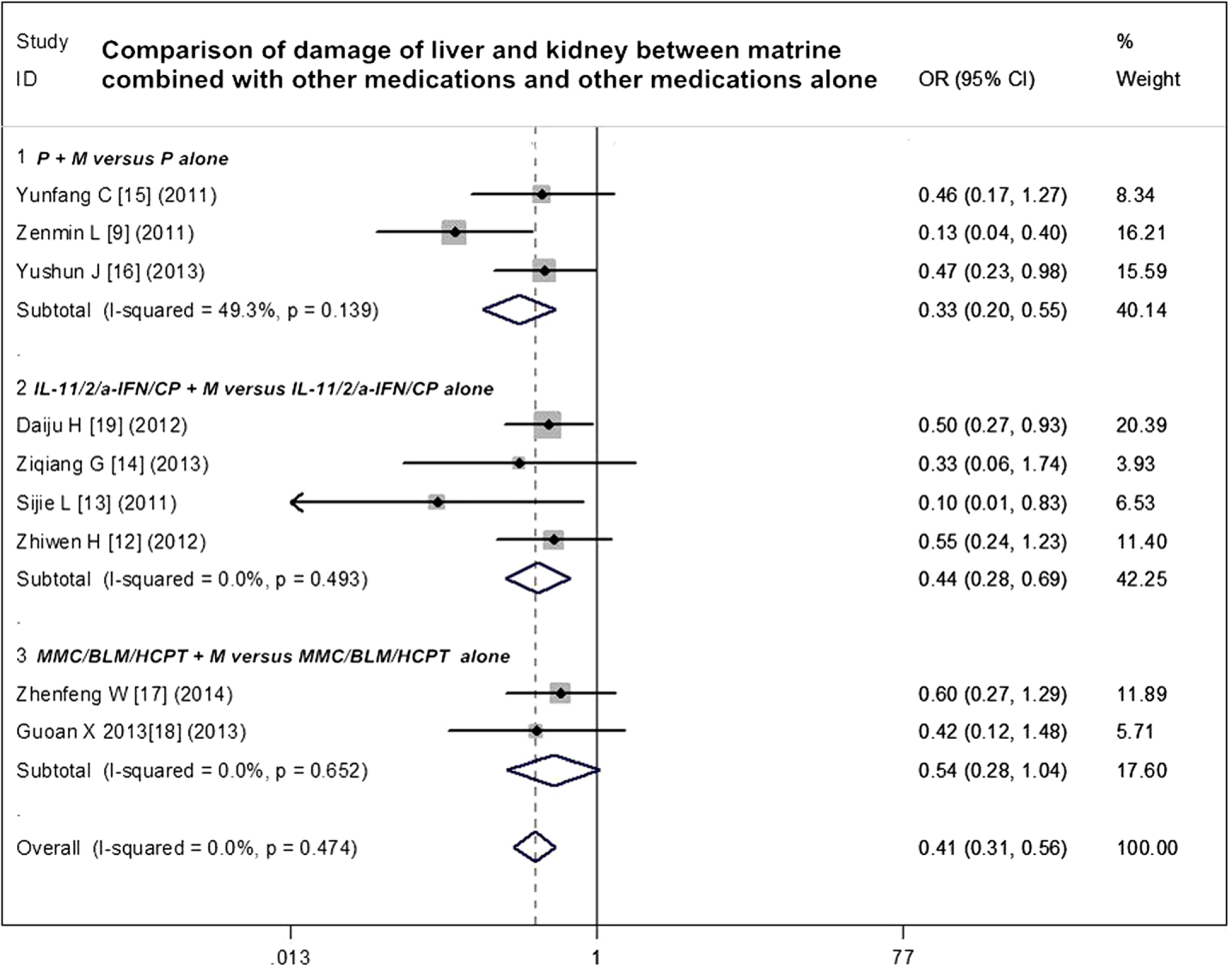
Comparison of damage of the liver and kidney between matrine combined with other medications and other medications alone. *ORR* overall response rate, *OR* odds ratio, *P* cisplatin, *M* murine, *IL-11* interleukin-11, *IL-2* interleukin-2, *а-IFN* а-interferon, *CP Corynebacterium parvum*, *MMC* mitomycin C, *BLM* bleomycin, *HCPT* hydroxycamptothecin

Included trials assessed seven serious AEs, the most common being gastrointestinal and hematologic diseases. Ten studies compared the myelotoxicity between matrine combined with other medications and other medications alone. The matrine combination arms had a lower incidence of myelotoxicity relative to the other medications arms (OR = 0.49, 95 % CI 0.37 to 0.64, *p* < 0.0001) (Fig. 5). Nine studies compared the damage of the liver and kidney between matrine combined with other medications and other medications alone. The result indicated that matrine combined with other medications had a lower incidence of the damage of the liver and kidney than other medications alone (OR = 0.41, 95 % CI 0.31 to 0.56, *p* < 0.0001) (Fig. 6). Eleven studies compared nausea/vomiting between matrine combined with other medications and other medications alone. Other common AEs including skin rash, nausea, vomiting, alopecia, nerve toxicity, and mucositis occurred with similar incidence in the two groups (*p* > 0.05).

### Analysis of publication bias

**Fig. 7:**
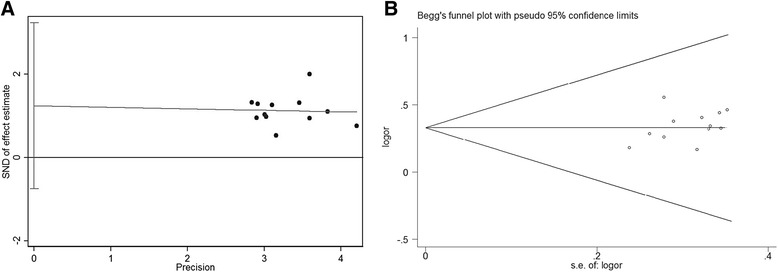
Assessment of publication bias. **a** Egger’s publication bias plot for the ORR of matrine combined with other medication and other medication alone; **b** Begg’s publication bias plot for the ORR of matrine combined with other medication and other medication alone

In the present study, the shape of the funnel plot appeared to be approximately symmetrical and suggested that publication biases may not have a significant effect on the results. The result of the Egger’s test was *t* = 1.39 (*p* = 0.195) (Fig. 7a), whereas that of the Begg’s test was std. dev. of score =14.58 (*p* = 0.086) (Fig. 7b). Therefore, both tests suggested that publication biases may not have a significant effect on the results.

## Discussion

MPE is a common clinical problem faced by many physicians, oncologists, and thoracic surgeons. Patients with MPE can be debilitated with dyspnea, decreased exercise tolerance, and impaired QOL. Median survival following the diagnosis of MPE ranges from 3 to 12 months, with lung cancer as the primary cancer demonstrating the shortest survival. The management options for MPE depend on several factors, including patient’s symptoms, performance status, underlying primary type, and the potential response to anti-neoplastic therapy. The overall aim is for the alleviation of symptoms and improved QOL. Matrine, a kind of alkaloid components found in the roots of *Sophora* species, is demonstrated to have anti-inflammatory, anti-virus, anti-fibrotic, and cardiovascular protective effects. They are recently proved to have anti-cancer potentials, such as inhibiting cancer cell proliferation, inducing cell cycle arrest, accelerating apoptosis, restraining angiogenesis, inducing cell differentiation, inhibiting cancer metastasis and invasion, reversing multidrug resistance, and preventing or reducing chemotherapy- or radiotherapy-induced toxicity when combined with other chemotherapeutic drugs [20]. *Sophora* root, which is a traditional herb medicine found in China, Japan, and some European countries, is the dried root of *Sophora flavescens* Aiton (Leguminosae) and includes matrine and oxymatrine, two major tetracyclo-quinolizindine alkaloids, as its primary components [20].

In recent years, some studies have reported on the efficacy and safety of matrine in the treatment of MPE. In this work, 12 reports of randomized trials were identified by searching from the start of each database up to January 2015. A significant benefit of matrine plus other medications in ORR was found (OR = 1.38, 95 % CI 1.17 to 1.64), translating into a 21 % absolute improvement. As follows, cisplatin + matrine versus cisplatin alone, biological agents (including IL-11, IL-2, а-IFN, and *Corynebacterium parvum*) + matrine versus biological agents alone, and other chemotherapeutic agents (including MMC, BLM, and HCPT) + matrine versus chemotherapeutic agents alone showed improvements of 16.1, 11.7, 24, and 20 % in ORR, respectively, which indicates that matrine combination therapy do better benefits in treating MPE via thoracic perfusion. Three reports analyzed that the time to pleural effusion relief of matrine combined with other medications (14.33 ± 1.20 months) in treating MPE was significantly longer than that of other medications alone (8.33 ± 0.88 months). Chest pain, commonly seen in pleural metastasis of malignant tumors, is typically localized to the side of the effusion and is described as dull and aching rather than non-malignant pleuritis. In present study, four trials indicated that the chest pain was significantly decreased by treating of matrine combined with other medications than that of other medications alone (*p* < 0.05). The relief of chest pain is an improvement of quality of life, and it is also an important aspect of treatment of patients. Matrine treatment has been shown to inhibit the proliferation of tumor cells in various cancers, including gastric cancer, breast cancer, hepatoma, colon cancer, melanoma, glioma, osteosarcoma, pancreatic cancer, and leukemia in a dose-dependent manner [20]. Resistance to apoptosis is a hallmark of cancer. Studies have reported that matrine exert anti-cancer effects by inducing apoptosis in different types of cancers. In non-small cell lung carcinoma (NSCLC), MT increases the phosphorylation of p38 and generates reactive oxygen species (ROS) in a dose- and time-dependent manner, which indicated that MT could activate p38 pathway and lead to a caspase-dependent apoptosis by inducing the generation of ROS [21].

Because QOL can be measured by various means, it is also quite easy to use it to measure and predict many variables during treatment. The benefit of chemotherapy in incurable cancers needs to be assessed directly through validated health-related QOL instruments rather than inferred from RRs, survival benefits, and other traditional endpoints. In the present study, 12 trials were enrolled in the assessment of QOL. A significant benefit of matrine plus other medications in the overall improvement rate of QOL (OR = 1.40, 95 % CI 1.18 to 1.66) was found, translating into a 24 % absolute improvement. Thus, the results showed that matrine can be used to relieve general side effects and improve patients’ QOL via pleural perfusion to cure MPE. The AEs found in the present analysis were mainly hematological reactions, diarrhea, toxicity of the liver and kidney, and nausea/vomiting, most of which were grade 1 or 2 and were well tolerated. The matrine combination arms had a lower incidence of myelotoxicity and dysfunction of the liver and kidney relative to the arms without matrine. And the incidence of nausea/vomiting of matrine combination arms was also significantly lower than that of other medications alone. The results supported that the matrine combination arms had a lower incidence of AEs compared with other medications alone, which indicates that matrine does has a impact on improving safety of chemotherapy and relieving general side effects. Overall, these results indicate that the potential benefit of matrine may be widely applicable to a patient population closely resembling clinical reality in advanced MPE.

In this review, the included studies were carefully assessed. A good clinical homogeneity was confirmed, and publication bias was not found according to the funnel plot analysis, the Egger’s test, and the Begg’s test. However, some deficiencies in the present work were found. First, the quality of subgroup analysis (age, sex, smoking, histology, and treatment status) according to the different agents (matrine plus other medications compared with other medications alone) was low because the subgroup data were only provided by a few trials. Second, some reports failed to report the method for concealment of allocation, blinding, and ITT. In addition, the partial reports comprise a small sample size, and some of the reports’ experimental control is not very balanced. Most of the included studies were published in Chinese, with heterogeneous data and analysis methods (e.g., the different scored scales were used to assess the life quality). Although such studies were reported to be of low quality, they still contain credible evidence pointing toward such new drugs. Clinical trials are expensive and difficult. Hence, these findings can help choose the most promising agents for study. However, matrine, as a new strategy, has still many issues to be resolved in further studies. Confirmation of these conclusions in rigorously controlled randomized trials is required before firm conclusions about this therapy can be drawn.

## Conclusions

The results showed that matrine combined with other medications was associated with higher ORR and superior QOL compared with other medications alone. Moreover, matrine combination therapy was shown to prolong the time to pleural effusion relief and decrease the incidence rate of chest pain and other AEs. Therefore, it indicates that matrine combination therapy exhibited superior efficacy and safety. The notable efficacy and activity of matrine in combination with other medications in treating MPE suggest that this regimen may have a value in the treatment of patients suffering from MPE, including those who cannot tolerate more aggressive therapies. However, confirmation of these conclusions in rigorously controlled randomized trials is required before firm conclusions about this therapy can be drawn.
